# A Novel Continuous-Flow PCR Microdevice Operated by a Single Heat Source

**DOI:** 10.3390/mi17070805

**Published:** 2026-06-30

**Authors:** Weining Song, Di Wu, Yutong Xing, Wenming Wu

**Affiliations:** 1 School of Artificial Intelligence and Information Engineering, East China University of Technology, Nanchang 330013, China; 201860174@ecut.edu.cn; 2 Changchun Institute of Optics, Fine Mechanics and Physics (CIOMP), Chinese Academy of Sciences, Beijing 100864, China; nymph_cc@163.com; 3 College of Physics Science & Technology, Hebei University, Baoding 071002, China; xingyutong@hbu.edu.cn

**Keywords:** single heat source, continuous-flow PCR (CF-PCR), PDMS thermal conduction block, Peltier effect (TEC), self-pressurized gas-diffusion micropump

## Abstract

This paper presents a constant-temperature, single-heat-source continuous-flow PCR (CF-PCR) microdevice that achieves stable thermal control for denaturation, annealing, and extension on a single platform. Key innovations include: (1) a metal-powder/PDMS thermal conduction block with trapezoidal geometry that generates a programmable temperature gradient and tunable residence times under one heat source; and (2) a thermoelectric cooler (TEC)-based Peltier system that creates distinct high- and low-temperature zones by co-optimizing the hot/cold side temperature difference, spacer material (92% alumina), and input voltage (3.6 V). A self-pressurized gas-diffusion micropump, enabled by a capillary quartz tube at the outlet, drives continuous sample flow without external actuation. The platform features three configurations: an on-chip zoned-heating design, an off-chip coiled-tube setup, and a battery-powered handheld system (727 g, 6 W, ~4 h runtime). Using CNC-machined and thermally bonded PMMA microchips with BSA passivation, the on-chip device achieves ~80% amplification efficiency relative to commercial instruments for H7N9 and pGEM-3Zf(+); the off-chip version reaches ~75%. The portable system yields HPV and RUBV amplification intensities comparable to benchtop devices. This approach provides a practical, scalable solution for “sample-in–answer-out” nucleic acid testing in point-of-care settings.

## 1. Introduction

For a long time, infectious and epidemic diseases caused by pathogenic microorganisms have posed a serious threat to human health and well-being and have been a major source of societal panic. As of 26 January 2021, the global COVID-19 pandemic had infected over 100 million people and caused more than 2.6 million deaths, constituting a global public health emergency. According to the World Health Organization (WHO), infectious diseases have claimed an average of over 17 million lives worldwide annually in recent years. Among these efforts, the rapid identification of pathogens is a core component in effectively controlling the spread of infectious diseases.

In recent years, polymerase chain reaction (PCR) technology—particularly real-time quantitative PCR (qPCR)—has become a cornerstone in pathogen detection due to its high sensitivity, speed, and ability to quantify microbial load in diverse water and clinical samples [1]. Meanwhile, gene chip technology and high-throughput sequencing have also advanced rapidly. Additionally, methods such as gel electrophoresis can be used to assess potential relationships among bacterial strains from different sources within the same outbreak event [2], playing a crucial role in source-tracing analyses—by establishing critical links between seemingly unrelated cases, thereby enabling precise identification of infection sources and facilitating effective disease prevention and control [3].

Conventional nucleic acid testing workflows typically involve multiple steps, including manual sample collection, sample transportation, laboratory-based nucleic acid extraction, preparation of detection reagents, loading into a nucleic acid analyzer, and product detection [4]. This process is not only time-consuming and operationally complex but also requires trained personnel to transfer samples between different instruments, greatly increasing the risk of contamination. However, outbreak sites or primary healthcare facilities often lack the necessary testing infrastructure and skilled personnel. Consequently, the development of integrated, all-in-one micro-devices for PCR-based detection has become a major research focus in recent years [5]. These compact, low-throughput microfluidic devices effectively minimize cross-contamination and offer the advantage of user-friendly operation, enabling even non-specialists to perform on-site testing—thus overcoming the spatial and temporal constraints of traditional laboratory settings.

Addressing these needs, this study focuses on the development of an integrated, automated PCR microdevice that combines sample preprocessing, PCR amplification, and product detection into a single platform for nucleic acid testing. The device features a compact size, portability, low cost, and ease of operation, enabling highly specific and sensitive automated detection of pathogens. Furthermore, leveraging microfluidic technology, this research explores a self-pressurized gas-diffusion micropump actuation mechanism and investigates both on-chip and off-chip PCR approaches for pathogen detection. A series of pathogen detection experiments were conducted, and the nucleic acid extraction performance was systematically evaluated using diverse samples, including bacteria and human hair. The ultimate goal is to achieve a fully automated “sample-in, answer-out” diagnostic workflow.

### 1.1. The Development of PCR Technology

Nucleic acids, as fundamental components of living organisms, are widely present in the cells of animals and plants, as well as in viruses and bacteria. Nucleic acid detection technologies enable direct determination of the genetic sequences of harmful organisms, allowing functional analysis at the levels of replication, transcription, translation, or structural organization, thereby providing a scientific basis for the diagnosis of human diseases [6,7,8]. DNA segments carrying genetic information are known as genes (or hereditary factors). Since the 1970s, techniques for the in vitro isolation and synthesis of DNA—integral to gene research—have attracted considerable attention. In 1971, Khorana and colleagues first proposed the concept of in vitro nucleic acid amplification: by denaturing DNA, hybridizing it with appropriate primers, and extending the primers using DNA polymerase through repeated cycles, specific genes (such as tRNA genes) could be synthesized [9]. In 1985, Kary Mullis, working at the Human Genetics Laboratory of PE-Cetus Corporation in the United States, invented the polymerase chain reaction (PCR) technique. PCR mimics the natural DNA replication process in vivo and requires only a DNA template, DNA polymerase, oligonucleotide primers, an appropriate buffer system, and precise thermal cycling to efficiently amplify specific DNA fragments in vitro [10]. This breakthrough was published in the journal Science, marking the official birth of PCR technology and rapidly earning widespread recognition within the life sciences community.

In 1976, Taiwanese scientist Alice Chien (Chien-Fu F. Chien) isolated the thermostable Taq DNA polymerase from *Thermus aquaticus*, a thermophilic bacterium found in Yellowstone National Park. In 1988, Saiki and colleagues incorporated this enzyme into the PCR system [11], significantly enhancing the reaction’s thermostability, specificity, and amplification efficiency. Since then, with continuous improvements in underlying theories and methodologies, PCR technology has been progressively adopted across diverse fields such as molecular biology, pathology, and medical diagnostics.

**Figure 1:**
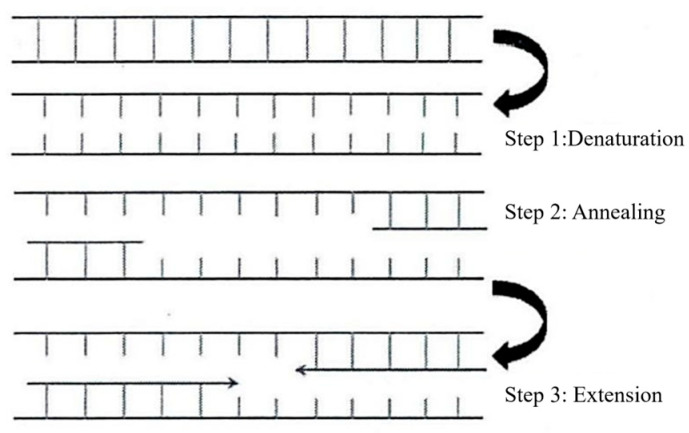
Schematic illustration of the PCR principle.

PCR is an in vitro nucleic acid amplification technique that specifically amplifies a target DNA template through an enzyme-mediated reaction guided by primers [12,13]. After approximately 30 thermal cycles of “denaturation–annealing–extension,” the target DNA fragment can be amplified up to a million-fold, facilitating subsequent detection or isolation of the gene of interest [14,15]. As illustrated in Figure 1, the fundamental principle involves using double-stranded DNA as a template; in the presence of the four deoxyribonucleoside triphosphates (dNTPs), sequence-specific primers bind to the 3′ ends of the template strands, and Taq DNA polymerase catalyzes the extension of complementary strands. Through repeated cycling, even a minute amount of initial template can undergo exponential amplification [16,17,18,19]. The theoretical copy number of the amplified product can be estimated by the following equation:

(1) $$Y=(1+X{)}^{n}$$

where Y represents the number of DNA copies after the n-th cycle, X is the average amplification efficiency per cycle, and n is the number of cycles [20].

The amplification buffer required for PCR provides an optimal environment for the DNA template. The four deoxynucleoside triphosphates (dNTPs) correspond to the nucleotide molecules of the four bases (A, T, G, C), and their quality and concentration also significantly affect PCR amplification efficiency. Prior to a PCR experiment, primers must be designed to enable specific amplification of the target DNA. Primer design typically requires sequences of 15–30 nucleotides in length [21]. During the extension phase of PCR, Taq DNA polymerase catalyzes the specific incorporation of the four dNTPs into the growing DNA strand complementary to the template, and its 5′→3′ polymerase activity is closely dependent on the Mg^2+^ concentration in the PCR mixture [22].

Since the thermal control performance of a PCR thermocycler directly determines the efficiency and accuracy of DNA amplification, its temperature control module must be rigorously calibrated before use. Owing to its high sensitivity and amplification efficiency, PCR technology has been widely applied to qualitative genetic analysis in numerous fields, including life sciences, clinical diagnostics, forensic identification, food safety, agronomy, archeology, and environmental protection, making it one of the most fundamental and essential techniques in molecular biology [23].

### 1.2. Research Significance of Continuous-Flow PCR (CF-PCR) Microdevices

Traditional PCR devices commonly employ microchamber-based architectures, which suffer from several limitations, including complex microvalve control, prolonged heating and cooling times due to a single thermal module, and difficulties in system integration. In contrast, continuous-flow PCR (CF-PCR) achieves thermal cycling and nucleic acid amplification by allowing reagents to flow continuously through microchannels that pass repeatedly among multiple heating zones held at fixed temperatures. This approach eliminates the need for complex microvalve systems and, owing to the seamless connectivity of the microchannels with upstream and downstream modules, facilitates the development of an integrated “sample-in–answer-out” diagnostic platform. Moreover, because the microchannel is positioned over multiple isothermal heating blocks, the overall PCR time is significantly reduced.

Currently, most conventional PCR instruments employ thermoelectric (Peltier-effect-based) cooling/heating modules for temperature control, achieving thermal cycling through bulk heating and cooling of the entire reaction chamber. In contrast, CF-PCR typically adopts a design in which the microchannel is positioned over multiple spatially separated, isothermally controlled heating blocks. A closed-loop control circuit independently regulates each zone to maintain the specific temperatures required for denaturation (~95 °C), annealing (~55–65 °C), and extension (~72 °C). As the reagent flows from the inlet through the microchannel and sequentially passes through these distinct temperature zones, one complete PCR cycle is accomplished. Although this configuration simplifies fluidic control, it still requires complex multi-channel temperature control circuitry and struggles to implement non-standard or dynamically varying temperature profiles, thereby limiting its operational flexibility.

To address the aforementioned challenges, this study focuses on a single-heater-based CF-PCR microdevice. This design employs only one isothermal heating element as the heat source, eliminating the need for multiple heating zones maintained at different stable temperatures. The required thermal cycling is achieved solely through dynamic modulation of this single heat source. This approach substantially simplifies the control system architecture and facilitates device miniaturization and integration. The ultimate goal of this research is to develop a standalone, compact CF-PCR microsystem: on one hand, by reducing the size of both the core device and its peripheral components to enhance portability; on the other hand, by incorporating a self-pressurized gas-diffusion micropump to replace conventional bulky external mechanical or constant-pressure pumps, thereby enabling autonomous, pump-free reagent transport.

Micropumps serve as a critical functional component in micro total analysis systems (μTAS), playing an essential role in integrated nucleic acid sample preparation and detection platforms designed for point-of-care testing (POCT). Since the development of the first true microelectromechanical systems (MEMS)-based micropump by Jan Smits and Harald Van Lintel in the 1980s [24,25], micropump technologies based on diverse actuation mechanisms have evolved into more than ten distinct types [26]. According to their energy supply method, existing micropumps can be broadly classified into two categories: active micropumps and passive (self-driven) micropumps. However, they typically require bulky external actuation hardware, making integration into compact, portable, and fully integrated diagnostic platforms challenging.

To overcome these limitations, researchers have recently developed various types of passive micropumps [27,28,29,30]. Unlike active counterparts, passive micropumps operate without external power supplies; instead, they autonomously induce fluid flow in microchannels by harnessing intrinsic physical or chemical mechanisms—such as capillary forces, evaporation, hydrostatic pressure, gas diffusion/permeation, chemical or enzymatic reactions, and biophysical effects—thereby significantly advancing the miniaturization and portability of microfluidic devices.

Common active micropumps also include commercially available syringe pumps, rotary pumps, and peristaltic pumps. Among these, syringe pumps—typically composed of one or multiple pistons—provide highly stable fluid actuation and are the most widely used in lab-on-a-chip (LOC) systems [31,32,33]. Rotary pumps [34] and peristaltic pumps [35] serve as alternative solutions in specific applications. In addition, researchers have developed various MEMS-compatible active micropumps: for instance, piezoelectric micropumps generate pumping action through the deformation of piezoelectric materials such as lead zirconate titanate (PZT) under an electric field [36,37,38]; pneumatic micropumps utilize sequential actuation of elastic microvalves fabricated from polydimethylsiloxane (PDMS) membranes, offering superior integrability into microsystems due to material flexibility and fabrication compatibility [39,40]; thermo-pneumatic micropumps also rely on membrane deflection but derive their driving force from localized heating of air or a secondary medium within a dedicated chamber, where thermal expansion generates the pressure required to propel fluid [41,42].

In this context, our study employs a self-pressurized gas-diffusion micropump based on gas permeation principles. This design enables continuous and stable sample transport without any external actuation, effectively balancing high system integration with field-deployable practicality. This paves the way toward a fully automated, field-deployable molecular diagnostic platform.

## 2. Materials and Methods

### 2.1. Principles and Operation of Temperature Control in Microdevices

#### 2.1.1. Temperature Control Scheme

##### Temperature Control Principle Based on PDMS Heat Transfer

The temperature control method based on PDMS heat transfer is primarily achieved through the temperature gradient formed by thermal resistance within the microreactor. By fixing the device onto a single heating element, the denaturation temperature required for PCR is attained. The height of the PDMS thermal block controls the temperature difference between the denaturation zone and the annealing/extension zone. The bottom surface of the PDMS thermal block is placed in contact with the heating element to reach the denaturation temperature, while the top surface corresponds to the annealing/extension temperature. The internal temperature gradient within the PDMS block is influenced by both natural convection heat transfer in the surrounding ambient space and conductive heat transfer inside the microdevice. Once the PDMS block reaches thermal equilibrium, the surface temperatures can be calculated using the following equation:

(2) $$\frac{t_{s}^{\prime }-t_{rt}}{t_{s}^{\prime \prime }-t_{s}^{\prime }}=\frac{R_{t}}{R_{m}+R_{f}}$$

where $R_{f}$ is the thermal resistance between the PCR microdevice and the heating plate, $R_{m}$ is the internal thermal resistance of the PDMS, and $R_{t}$ is the thermal resistance due to natural convection between the top surface of the PDMS and the ambient air., $t_{s}^{\prime }$ denotes the temperature at the top surface of the PDMS, $t_{s}^{\prime \prime }$ denotes the temperature at the bottom surface of the PDMS (which is approximately equal to the temperature of the heating plate), and $t_{rt}$ represents the room temperature. At steady state, the heat conducted through the chip ($Q_{c}$) balances the heat transferred from the chip to the surrounding air via natural convection ($Q_{a}$), i.e.,

(3) $$Q_{c}=Q_{a}$$

According to Fourier’s law, $Q_{c}$ can be expressed as:

(4) $$Q_{c}=\lambda \frac{t_{s}^{\prime \prime }-t_{s}^{\prime }}{l}A$$

where λ is the thermal conductivity of the PDMS material, l is the heat transfer distance within the PDMS (i.e., the thickness of the PDMS), and A is the cross-sectional area for heat transfer through the PDMS. The heat transfer rate due to natural convection in air can be calculated using the following equation and expressed as:

(5) $$Q_{a}=\alpha (t_{s}^{\prime }-t_{rt})$$

where α is the thermal conductivity of air.

Based on the above equations, the temperature gradient within the PDMS can be tailored to meet the desired thermal requirements by adjusting the material dimensions (cross-sectional area and heat transfer distance) and selecting appropriate materials (thermal conductivity).

In this experiment, copper powder was added to PDMS to fabricate a metal-powder–PDMS composite, and different thermal conductivities were achieved by varying the copper powder concentration. As shown in Table 1, all hybrid PDMS-based thermal control modules were fabricated with identical dimensions: 60 mm in length, 10 mm in width, and 15 mm in height. Taking into account the intrinsic thermal conductivity of the PMMA microreactor chip itself, three thermal control modules—containing 4% copper, 15% copper, and pure copper—were ultimately selected for the three-temperature-zone on-chip PCR microdevice. By setting the heating plate temperature to 120 °C and placing each of the three thermal modules, respectively, over the denaturation, extension, and annealing regions, the top surfaces of the modules stabilized at temperatures that fulfill the requirements for PCR amplification.

**Figure 2:**
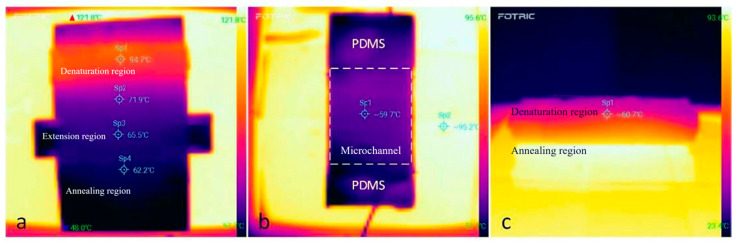
Infrared thermography of the PCR process: (**a**) on-chip PCR microdevice top surface; (**b**) off-chip PCR microdevice top surface; (**c**) off-chip PCR microdevice side view.

For the off-chip PCR microdevice, it is sufficient to set the heating plate temperature to 95 °C to achieve the required denaturation temperature in the denaturation zone. Depending on specific experimental requirements, the temperature at the top surface of the annealing zone can be fine-tuned by adjusting the height of the PDMS thermal control module. Additionally, the two inclined sidewalls of a trapezoidal PDMS thermal module can serve as the extension zones, enabling precise and stable control over the entire PCR thermal cycling process. As shown in Figure 2, the temperature distribution captured by an infrared thermal camera demonstrates this effect. By leveraging the thermal conduction properties of the PDMS thermal control modules, both on-chip and off-chip PCR systems can achieve the necessary temperatures for denaturation, extension, and annealing stages, ensuring excellent temperature uniformity and stability throughout each phase.

##### Temperature Control Principle Based on the Peltier Effect

A thermoelectric cooler (TEC) is a heat-transfer device that exploits the Peltier effect. When an electric current passes through a thermocouple pair composed of an n-type and a p-type semiconductor, heat is transferred from one junction to the other, creating a temperature difference and resulting in distinct hot and cold sides.

**Figure 3:**
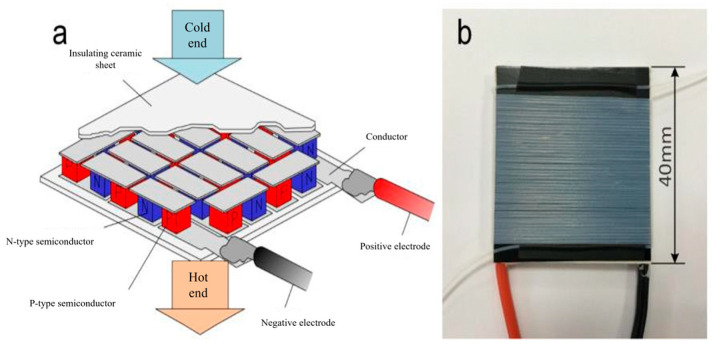
Peltier effect of the thermoelectric cooler (TEC) and schematic illustration of the PCR microdevice: (**a**) schematic diagram of the TEC structure; (**b**) photograph of the PCR microreactor based on the TEC Peltier effect.

As shown in Figure 3a, each micro-module consists of one n-type and one p-type semiconductor. The Peltier effect occurs at the junction between these two dissimilar semiconductor materials. When a direct current flows through this junction, a temperature difference is generated.

The amount of Peltier heat generated per unit time by each micro-module is given by:

(6) $$\dot{Q}=(\Pi_{P}-\Pi_{N})I$$

where $\Pi_{P}$ and $\Pi_{N}$ are the Peltier coefficients of the p-type and n-type semiconductor materials, respectively, and I is the electric current flowing through the conductor.

(7) W=ΠIt

#### 2.1.2. Sample Introduction Method

This system requires no external pumps or valves; instead, it generates a stable pressure differential by compressing a sealed gas chamber and, in conjunction with flow-resistance regulation within microchannels, enables automatic, uniform, and bubble-free sample injection.

Its working principle is as follows: the fluid flow velocity within the microchannel is determined by the dynamic balance between the pressure gradient and the flow resistance. The flow resistance can be described by the Hagen–Poiseuille equation:

(8) $$P_{r}=\frac{8\mu lv_{l}}{r^{2}}$$

where $P_{r}$ denotes the flow resistance, l is the length of the liquid segment, $v_{l}$ is the fluid velocity, μ is the fluid viscosity, and r is the radius of the microchannel. As the sample continuously flows into the microchannel driven by the pressure difference ΔP, the liquid segment length (l) gradually increases; if the flow velocity remains constant, the flow resistance correspondingly increases. When $\Delta P=P_{r}$ is satisfied, the system reaches a steady-state flow.

The pressure difference ΔP is generated by pressurizing a syringe. The specific sample-loading procedure is as follows: First, 40 μL of sample reagent is dispensed into the bottom of the syringe using a micropipette. Next, the syringe is connected to the PTFE microtubing of the PCR microreactor. Prior to sample introduction, the distal end of the capillary PTFE tubing is clamped shut with a clip to ensure the system is completely sealed. Then, the syringe plunger is steadily pushed from the 20 mL mark to the 10 mL mark and secured in place with a wire, thereby compressing the air inside the device and establishing a pressure environment higher than atmospheric pressure. At this stage, the internal pressure within the microdevice exceeds ambient atmospheric pressure, creating flow resistance at the clamped location, but no fluid flow occurs yet.

Assuming the gas inside the device behaves as an ideal gas, the following relationship holds:

(9) nRT=PV

Substituting the initial and pressurized states yields:

(10) $$nRT=P_{atm}\left(V_{c}+V_{s}\right)$$

(11) $$nRT=\left(P_{atm}+\Delta P\right)\left(V_{c}+V_{s}-\Delta V\right)$$

where n is the amount of gas (in moles), T is the thermodynamic temperature, R is the ideal gas constant, $V_{c}$ and $V_{s}$ are the gas volumes in the microchannel and the syringe, respectively, $P_{atm}$ is the atmospheric pressure, and ΔV is the change in gas volume caused by the movement of the syringe plunger.

Upon removing the clamp at the distal end, the pressure difference ΔP acts across the two ends of the liquid column, disrupting the pressure equilibrium and initiating sample flow. Under steady-state conditions, the total pressure difference equals the sum of the flow resistances of all segments:

(12) $$\Delta P=\frac{8\mu_{0}l_{0}v_{0}}{r_{0}^{2}}+\frac{8\mu_{c}l_{c}v_{c}}{r_{c}^{2}}+\frac{8\mu_{q}l_{q}v_{q}}{r_{q}^{2}}$$

where

$\mu_{0},\mu_{c},\mu_{q}$ are the effective viscosities of the sample in the connecting silicone tubing, the main microchannel, and the capillary quartz tube, respectively;

$l_{0},l_{c},l_{q}$ are the lengths of the liquid column in the corresponding segments;

$v_{0},v_{c},v_{q}$ are the flow velocities in each segment;

$r_{0},r_{c},r_{q}$ are the inner radii of the thick-walled silicone tube, the microchannel, and the capillary quartz tube, respectively.

Since the plunger position is fixed, ΔP can be regarded as constant; consequently, under steady-state conditions, the flow velocities in all segments also stabilize, enabling long-term, stable, and uniform fluid delivery. Moreover, the flow rate can be effectively controlled by adjusting the geometric parameters of the microchannels (e.g., increasing the radius or shortening the length).

In practical operation, after securing the plunger, the syringe is placed vertically, allowing the sample reagent to automatically flow into the microchannel under the driving force of the pressure difference. Once sample loading is complete, the microreactor is ready for thermal cycling. After 40 full thermal cycles—comprising denaturation, annealing, and extension steps—the target DNA fragment is efficiently amplified.

It should be noted that the above analysis is based on the ideal gas assumption and does not account for non-ideal effects of real gases; therefore, minor discrepancies may exist between theoretical predictions and experimental results. Nevertheless, this model accurately captures the core operating mechanism of the system and provides reliable technical support for a passive, integrated microfluidic PCR platform.

### 2.2. Fabrication of the Microdevice

#### 2.2.1. On-Chip CF-PCR Microdevice with Single Heat Source and Zoned Heating

The on-chip CF-PCR microdevice primarily consists of a PCR microreactor chip, metal-powder–PDMS thermal conduction blocks, and heating plates.

First, a continuous-flow microchannel is designed according to the required thermal cycling profile of the sample reagent, ensuring that the reagent passes sequentially through designated temperature zones as it flows through the channel. Correspondingly, multiple metal-powder–PDMS thermal conduction modules with identical heights but varying metal-powder ratios are fabricated to match the specific temperature requirements of each thermal cycle stage, so that the surface temperature of each module aligns precisely with the target reaction temperature.

These thermal modules are then mounted onto a constant-temperature heating plate, and the PCR microreactor chip is placed on top of the modules, with the microchannels aligned to their respective temperature zones. Black adhesive tape is applied to the top surface of the PDMS microchip to facilitate temperature monitoring of individual reaction zones using an infrared thermal camera. Finally, a portable power module is used to control the heating plate. Once thermal equilibrium is reached across all temperature zones of the chip, the PCR amplification experiment can be initiated.

#### 2.2.2. Off-Chip CF-PCR Microdevice with a Single Heat Source

**Figure 4:**
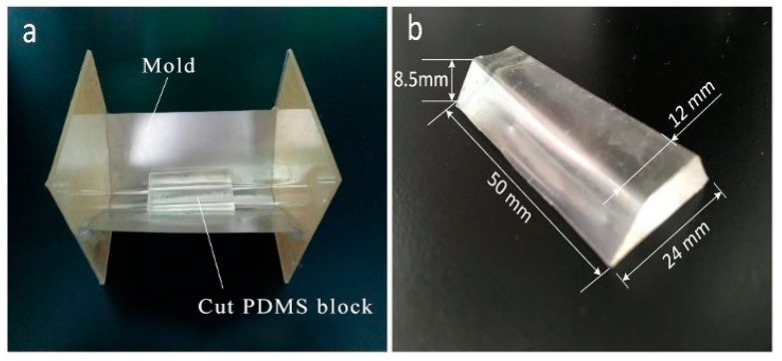
PDMS thermal conduction block and its mold. (**a**) Trapezoidal copper mold used for fabricating the PDMS thermal conduction block; (**b**) dimensions of the PDMS thermal conduction block employed in the experiment.

The CF-PCR microdevice based on PDMS thermal conduction primarily consists of a self-pressurized gas-diffusion micropump utilizing capillary quartz tube flow resistance, a heating plate, capillary PTFE tubing (inner diameter = 0.3 mm, outer diameter = 0.6 mm), and a PDMS thermal conduction block, as shown in Figure 4.

**Figure 5:**
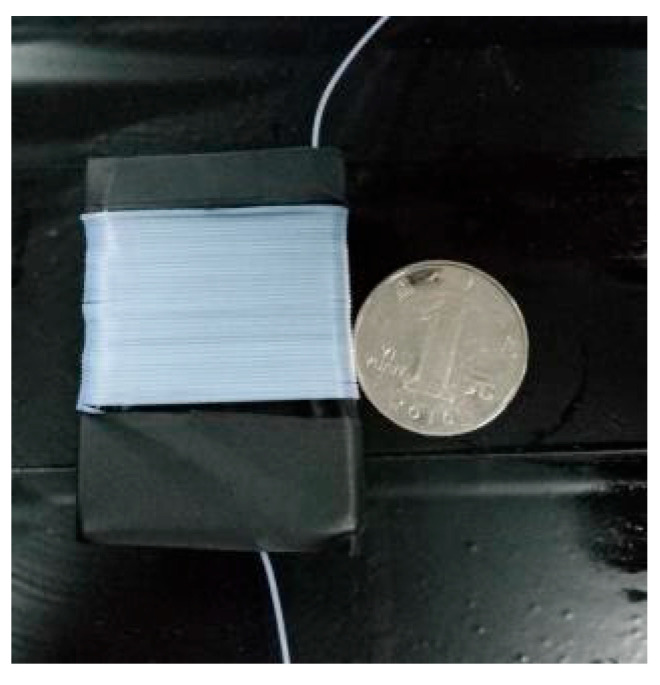
Physical image of PCR microreactor.

Prepared PDMS prepolymer is poured into a trapezoidal copper mold (as illustrated in Figure 5) and cured for one hour in a vacuum oven at 70 °C, resulting in a PDMS block with a top surface width of 24 mm, a bottom surface width of 12 mm, a height of 8.5 mm, and a length of 50 mm. The height of the PDMS block can be freely adjusted according to the temperature requirements of PCR, thereby enabling vertical tuning of the corresponding thermal gradient.

As shown in Figure 5, the prepared PDMS thermal conduction block is wrapped with black PVC tape to facilitate temperature measurement using a thermal infrared camera. The capillary PTFE tubing is then coiled around the PDMS block for 40 turns, with each turn representing one thermal cycle of the PCR. Additionally, a capillary quartz tube with an inner diameter of 25 μm and a length of 10 cm is connected at the outlet of the microchannel, completing the assembly of the PCR microreactor.

The PDMS microdevice is placed on the surface of the heating plate and secured with tape. A simple temperature control module (comprising a battery and a miniature voltage regulator) is then used to heat the plate to 95 °C, and the system is held at this temperature for 10 min. Once thermal equilibrium is reached, an infrared thermal camera is used to verify that the top surface of the PDMS microdevice has attained the annealing temperature required for PCR, after which the PCR experiment can be initiated.

#### 2.2.3. Off-Chip CF-PCR Microdevice Based on the Peltier Effect

By leveraging the Peltier effect of a thermoelectric cooler (TEC), a portable, handheld CF-PCR amplifier was developed, featuring a self-pressurized gas-diffusion micropump—connected at the outlet to an impermeable capillary quartz tube—and a constant single-heat-source temperature control scheme. The device weighs only 727 g (including an independent power module) and consumes merely 6 W of power.

**Figure 6:**
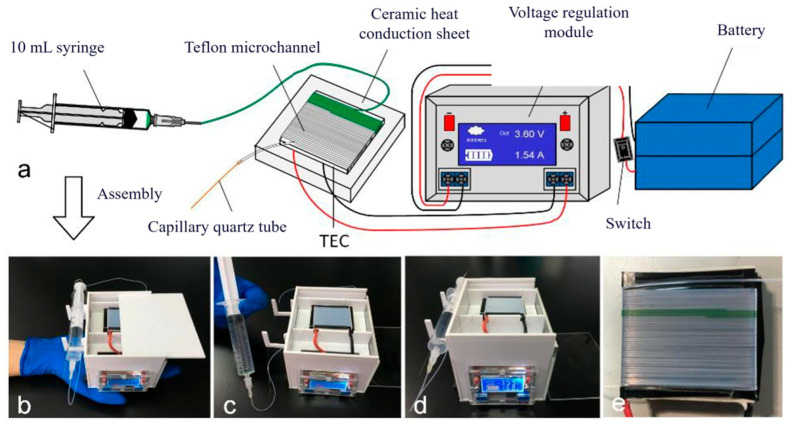
Portable hand-held continuous-flow PCR microdevice. (**a**) Schematic illustration of the microdevice; (**b**–**d**) photographs of the assembled microdevice; (**e**) image showing reagent flow within the microchannel.

As shown in Figure 6, the portable handheld CF-PCR amplifier measures 115 mm × 85 mm × 80 mm. It consists of two series-connected 3.7 V polymer lithium-ion batteries (12,000 mAh), a voltage regulation module, a switch, a ceramic heat-conducting plate (92% alumina ceramic), a thermoelectric cooler (TEC1-12712, 40 mm × 40 mm × 3.3 mm), a 10 mL syringe, capillary PTFE tubing (inner diameter = 0.3 mm, outer diameter = 0.6 mm), a piece of wire, a 27 G needle, and a 15 cm-long capillary quartz tube (inner diameter = 25 μm). The housing is made of PMMA.

The TEC1-12712 thermoelectric cooler is commonly used in water dispensers or refrigerators as a semiconductor cooling device, with a maximum temperature difference of 62 °C. Therefore, by adjusting the input voltage, the cold and hot sides can be stabilized at suitable temperatures for PCRs. Moreover, the surface temperature of the TEC is influenced not only by the input voltage but also by the thermal conductivity of the gasket material. Thus, to determine appropriate amplification conditions, this experiment selected three materials with suitable thermal conductivities for voltage gradient experiments: 92% alumina ceramic, silicon carbide ceramic, and 304 stainless steel. These materials were all of identical dimensions: 50 mm × 50 mm × 10 mm.

To meet the temperature requirements for the denaturation stage, the experiment utilized three different gaskets and various voltages to monitor the temperature in the high-temperature zone, while recording the temperatures in the low-temperature zone during gradient experiments. Ultimately, 92% alumina ceramic was chosen as the gasket material for this microdevice.

### 2.3. Experimental Sample Preparation

#### 2.3.1. Plasmid Sample Preparation

Plasmids and primers for H7N9 avian influenza, pGEM-3Zf (+), Human papillomavirus (type 49), and RUBV were purchased from Genewiz. According to the manufacturer’s instructions, DNA templates were diluted to various concentrations (e.g., 10^7^ copies/μL, 10^6^ copies/μL, 10^5^ copies/μL, 10^4^ copies/μL, etc.), and primers were diluted to a working concentration of 10 μM for subsequent use.

#### 2.3.2. PDMS Fabrication Procedure

Take the required amount of polydimethylsiloxane (PDMS) and curing agent, mix them in a ratio of 10:1, put them in a disposable weighing dish and mix well. The weighing dish was then placed in a plastic vacuum desiccator at a negative pressure of 12 psi for 20 min to allow air bubbles to escape from the inside. After all bubbles disappear, take it out and set aside.

#### 2.3.3. Fabrication of PMMA Microfluidic Chips

(1) PMMA chip design and fabrication

**Figure 7:**
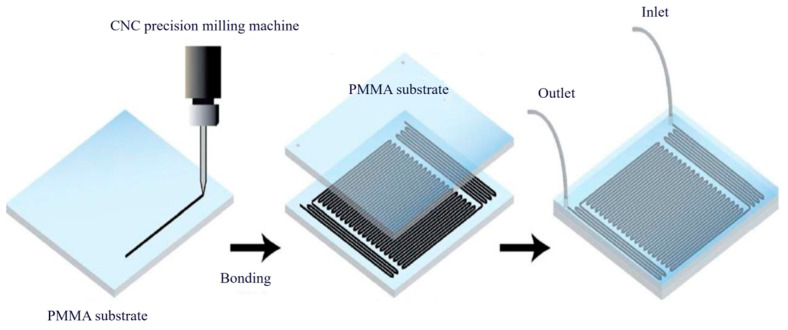
The schematic diagram of PMMA microchip processing.

The fabrication of POCT microfluidic chips generally involves two main steps: (1) forming the microstructures on the chip, and (2) bonding the chip layers. Using a mechanical machining approach, microchannels were directly milled into PMMA substrates with a small-diameter (0.2 mm) end mill via a CNC precision engraving machine. The microchannel design is illustrated in Figure 7.

(2) PMMA Chip Bonding Method

POCT microfluidic chips typically consist of a substrate and a cover plate. After microchannels are fabricated on the substrate, they must be bonded to the cover plate to form enclosed fluidic pathways. Several factors must be considered when bonding POCT microfluidic chips:

① Deformation of microchannels: Changes in microchannel dimensions directly alter internal fluidic pressure, thereby affecting flow velocity. However, bonding methods such as thermal bonding require the application of high temperature and pressure, which can compress and deform the microchannels, compromising device performance.

② Surface wettability: The wettability of microchannel surfaces influences liquid flow behavior. Bonding approaches like adhesive film bonding or oxygen plasma-assisted bonding often introduce intermediate layers or modify the channel surface chemistry, potentially altering flow dynamics and consequently affecting analytical results.

③ Bonding strength: Bonding strength is a critical indicator of bond quality. Higher bonding strength enhances the chip’s durability and reliability, thereby extending its operational lifetime.

In this study, thermal bonding was employed to seal the microfluidic chips. The thermal bonding process is straightforward: the substrate and cover plate are heated in a hot-press machine to a temperature near the glass transition temperature of the material (approximately 90 °C) and subjected to a certain pressure. Bonding is completed after a period of simultaneous heat and pressure retention. The elevated temperature and pressure enhance intermolecular interactions at the bonding interface, thereby improving bond strength. The specific hot-pressing steps are as follows:

(1) Cut the PMMA sheets into small pieces measuring 90 mm × 60 mm × 2 mm (length × width × thickness). Place the cut PMMA pieces into an ultrasonic cleaner and clean them for 10 min. After cleaning, dry the PMMA pieces with compressed air, then place them in an oven at 50 °C for 5 h before use.
(2) Place the glass pressing plates, substrate, cover plate, and another glass pressing plate onto the hot-press machine’s stage in a top-to-bottom sequence. Due to the low surface roughness of the glass plates, direct contact with the PMMA material prevents the transfer of mechanical machining marks from the press platens onto the material surface.
(3) Turn on the hot-press machine and set the temperature of both the upper and lower heating plates to 90 °C. Apply pressure to the PMMA sheets at a rate of 0.02 MPa/s. Once the pressure reaches 2.0 MPa, maintain this pressure for 15 min. To minimize elastic recovery (spring-back) of the PMMA during cooling, turn off the heating module after bonding is complete, allow the system to cool naturally to approximately 60 °C, then raise the press head and remove the bonded chip.

(3) PMMA Chip Surface Treatment Method

Due to the large internal surface area of capillary microchannels used as PCR microreactors, surface adsorption can occur, leading to reduced reaction efficiency. To mitigate this issue, the microchannels can be passivated with bovine serum albumin (BSA) prior to PCR, thereby preventing non-specific adsorption onto the channel walls.

Accurately weigh 0.05 g of crystalline bovine serum albumin (BSA) on an analytical balance and transfer it into a small beaker. Add a small amount of distilled water to dissolve the BSA, then transfer the solution into a 100 mL volumetric flask. Rinse the beaker several times with small portions of distilled water, and combine all rinses into the volumetric flask. Finally, dilute to the 100 mL mark with distilled water to prepare a 0.5 mg/mL BSA solution. Using a syringe, draw up 5 mL of the BSA solution and inject it into the microchannels of the PCR chip until the channels are completely filled. Allow the chip to incubate for 4 h, after which the BSA solution is flushed out from the microchannels.

### 2.4. Setting of Experimental Conditions

The composition of the PCR mixture (20 μL total volume) and the PCR cycling program are as described in Table 2, Table 3, Table 4, Table 5, Table 6 and Table 7.

### 2.5. Reaction Procedure

For H7N9 avian influenza and pGEM-3Zf(+) plasmid vector:

95 °C (30 s) ⟷ 60 °C (50 s)

40 cycles

95 °C (5 min)                       

95 °C (30 s) ⟷ 60 °C (50 s)

40 cycles

Perform gel electrophoresis to analyze the PCR products. The experiment requires 1× TBE buffer, which can be prepared by diluting the 10× TBE buffer purchased from Phygene Biotechnology tenfold. The detailed procedure for gel electrophoresis is as follows:

(1) Preparation of agarose solution: Accurately weigh 0.4 g of agarose and place it in an Erlenmeyer flask. Add 20 mL of 1× TBE buffer, mix thoroughly, and heat in a microwave for 1 min to completely dissolve the agarose. Seal the flask opening with breathable sealing film and a rubber band to prevent excessive evaporation of the liquid.

(2) Gel casting: Place a clean gel tray horizontally. When the agarose solution has cooled to 60–70 °C, add 2 μL of Gel-Green dye, mix gently, and carefully pour the solution into the gel tray. Immediately insert a comb to form sample wells. Gel-Green binds to DNA and produces visible bands under UV illumination. Allow the gel to solidify for approximately 30 min, then carefully remove the comb. Place the gel tray into a horizontal electrophoresis chamber and fill the chamber with 1× TBE buffer as the running buffer.

(3) Sample loading: Carefully load 5 μL of the PCR amplification product into the wells of the agarose gel, taking care not to damage the well walls.

(4) Electrophoresis: Immediately apply voltage after sample loading. Run electrophoresis at a constant voltage of 100 V for 40 min. Since DNA molecules carry a negative charge, the bands will migrate from the negative electrode toward the positive electrode.

(5) Imaging: After electrophoresis, visualize the DNA bands using a multifunctional UV transilluminator. Adjust camera settings such as exposure time, then capture and save the image for analysis.

## 3. Results

### 3.1. On-Chip CF-PCR Microdevice for Pathogen Detection

**Figure 8:**
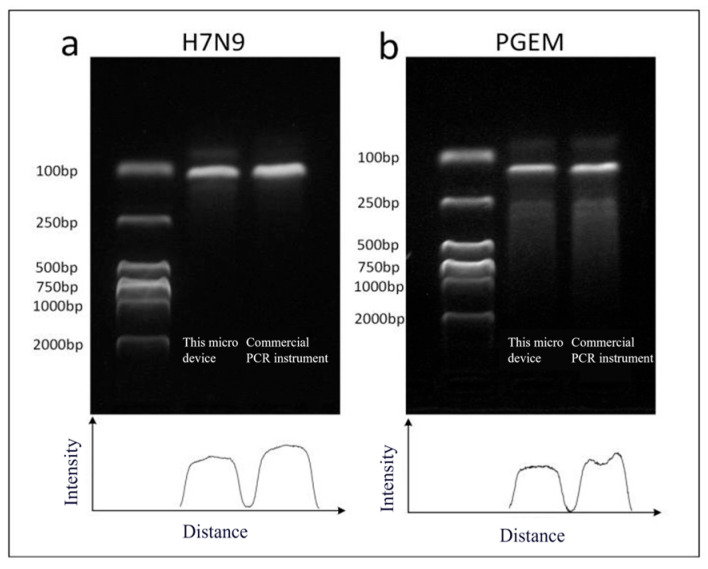
Electrophorogram and intensity diagram of the on-chip PCR.

**Figure 9:**
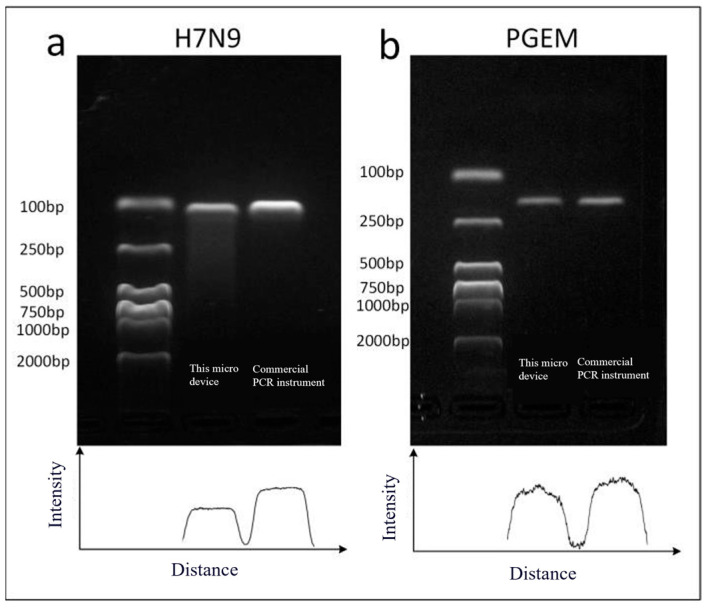
This is an electrophorogram and intensity diagram of the off-chip PCR. Panels are listed as follows: (**a**) H7N9 avian influenza; (**b**) pGEM-3Zf (+) DNA fragment.

To verify the amplification stability of the on-chip CF-PCR microdevice, two primer–template pairs were selected: a 116 bp gene fragment from the H7N9 avian influenza virus and a 137 bp gene fragment from Escherichia coli pGEM-3Zf (+). For each primer–template pair, three replicate experiments were conducted, and the optimal results are shown in Figure 8 and Figure 9. These results were compared with those obtained using a commercial thermal cycler, which was programmed with the following cycling conditions: 95 °C for 15 s, followed by 60 °C for 30 s, repeated for 40 cycles. The flow rate of the sample reagent within the microchannel was measured to be approximately 40 s per cycle, with residence times of approximately 10 s and 20 s in the high-temperature and low-temperature zones, respectively.

By utilizing multiple highly identical PDMS temperature control modules to partition and heat the chip, different regions of the chip can be maintained at different temperatures, thereby meeting the temperature requirements for the denaturation, annealing, and extension stages of PCR. The PDMS temperature control modules are made from a mixture of metal powder and PDMS, and by altering the ratio of each component, various combinations can be easily achieved to realize complex temperature cycling. Additionally, adjustments to the height of the PDMS temperature control modules can modify the temperature of their upper surfaces. This method does not require expensive temperature control equipment, has a simple structure, is easy to implement, and offers good economic practicality, making it convenient for large-scale promotion and use.

As can be seen from the gel electrophoresis results, the amplification intensity of the target gene obtained using the on-chip CF-PCR microdevice is comparable to that achieved with a commercial PCR instrument. Although adsorption onto the inner surface of the microchannels slightly reduces PCR efficiency—resulting in slightly weaker electrophoretic bands compared to those from the commercial PCR instrument—this effect is unavoidable in CF-PCR. Based on a comprehensive analysis of the target gene amplification results shown in Figure 8, the average amplification efficiency of the CF-PCR microdevice is approximately 80% that of the commercial PCR instrument, which is reasonably close to the performance of the commercial system.

### 3.2. Off-Chip CF-PCR Microdevice for Pathogen Detection Based on PDMS Heat Transfer

**Figure 10:**
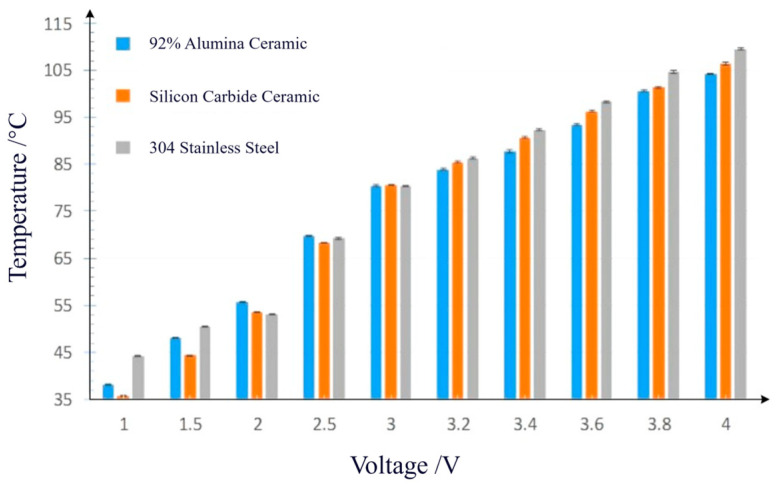
The temperature of high temperature zone under different voltage and plate material.

For each primer–template pair, three replicate experiments were performed, and the best results are shown in Figure 10. These results were compared with those obtained from a commercial thermal cycler, which was programmed with the following cycling conditions: 95 °C for 15 s, followed by 60 °C for 30 s, repeated for 40 cycles. The flow rate of the sample reagent through the microchannel was measured to be approximately 42 s per cycle, with residence times of approximately 12 s and 20 s in the high-temperature and low-temperature zones, respectively.

Figure 9a and Figure 10 show the amplification results of the target genes using this CF-PCR microdevice and a commercial thermal cycler, respectively. Based on a comprehensive analysis of the amplification results in Figure 10, the average amplification efficiency is approximately 75% of that of the commercial PCR instrument, which is fairly close to the performance of the commercial system. Additionally, by employing a single constant-temperature heating plate to heat the chip, instead of multiple heating plates maintained at different temperatures, the structure of the temperature control system is significantly simplified.

The design of the PDMS temperature control module’s shape is one of the advantages of this microdevice. Traditional cuboid shapes have equal durations for denaturation and annealing stages. In contrast, the improved trapezoidal PDMS heat-conducting block adjusts the allocation of durations for each stage of the PCR temperature cycle through its external structure. This adjustment ensures that the sample reagents experience different durations in various temperature zones, allowing for precise control of reaction times by altering the dimensions of the upper and lower surfaces of the heat-conducting block. Moreover, the temperature in the annealing zone can be set by adjusting the height of the PDMS heat-conducting block; the taller it is, the longer the annealing time. This method enables PCR amplification of different samples using a single PDMS module with relatively high reaction efficiency, simple operation, compact size, and ease of integration with upstream and downstream modules.

### 3.3. Temperature Control and Pathogen Detection in the Peltier Effect-Based CF-PCR Microdevice

#### 3.3.1. Spacer Selection

To achieve a miniaturized and portable CF-PCR microdevice, a handheld continuous-flow PCR amplifier based on the Peltier effect using thermoelectric coolers (TECs) was developed. The system weighs only 727 g (including an independent power module) and consumes an average power of just 6 W during operation. When fully charged, it can operate continuously for up to 4 h. During operation, the user turns on the switch and adjusts the miniature voltage module to 3.6 V, followed by a 10 min warm-up period. Prior to amplification, sample loading is performed by pushing the syringe plunger from the 10 mL mark to the 6 mL mark and securing it in place with a wire. The sample reagent circulates through the microchannel at a flow rate of 8.5 μL/min, completing one loop approximately every 60 s—conditions that satisfy the requirements for PCR amplification.

This microdevice can achieve most of the required conditions for PCR by adjusting parameters such as the input voltage and the thickness of the spacer material. To determine optimal amplification conditions, three materials with suitable thermal conductivities were selected for voltage gradient experiments: 92% alumina ceramic, silicon carbide ceramic, and 304 stainless steel. As shown in Figure 10 and Table 8, when 92% alumina ceramic was used as the spacer and the applied voltage was set to 3.6 V, relatively favorable PCR conditions were achieved.

#### 3.3.2. Pathogen Detection Results

**Figure 11:**
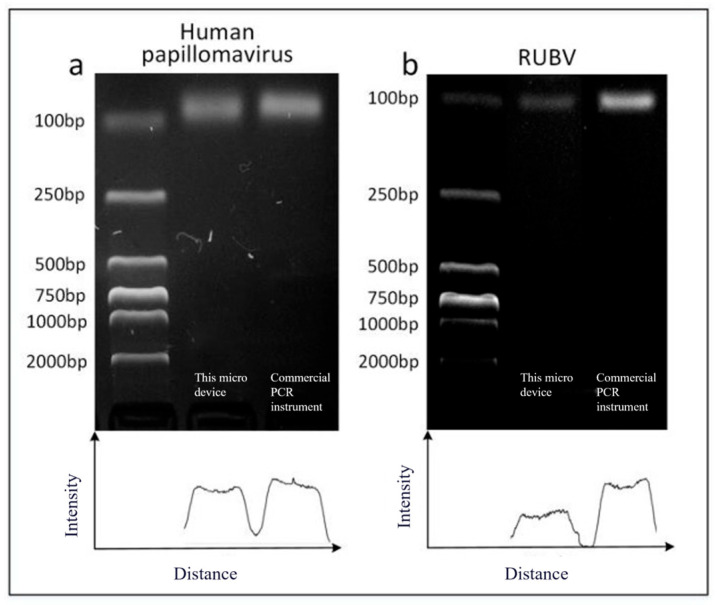
Electrophorogram and intensity diagram of the off-chip PCR amplification results using a microdevice. (**a**) Human papillomavirus; (**b**) RUBV fragment.

To verify the amplification stability of the portable handheld continuous-flow PCR amplifier, two primer–template pairs were selected: a 75 bp gene fragment of Human Papillomavirus (HPV) and an 88 bp gene fragment of Rubella virus (RUBV). For each primer–template pair, three replicate experiments were performed, and the best results are shown in Figure 11. These results were compared with those obtained using a commercial thermal cycler, which was programmed with the following cycling conditions: 95 °C for 15 s (denaturation), 60 °C for 30 s (annealing/extension), for a total of 40 cycles. In the microdevice, the sample reagent flowed through the microchannel at an average speed corresponding to approximately 40 s per cycle, with residence times of about 10 s in the high-temperature zone and 20 s in the low-temperature zone, respectively.

The gel electrophoresis detection results show that the amplification intensity of the target genes obtained using this microdevice is quite close to that obtained with a commercial PCR machine. Compared with other CF-PCR microdevices, firstly, this device uniquely utilizes the temperature difference between the hot and cold ends of a thermoelectric cooler (TEC) under isothermal conditions for PCR, achieving miniaturization and portability. Secondly, while other microdevices typically use AC power to supply electricity to heating plates (or heating stages), this microdevice employs a lithium battery as its power source, thereby eliminating the need for external power supply. This feature allows for conducting PCR experiments outdoors without access to an external power source, giving it a significant potential advantage in the field of on-site and immediate detection.

## 4. Discussion

This study developed a continuous-flow PCR (CF-PCR) microdevice based on a single heat source, which achieves efficient and portable nucleic acid amplification through the synergistic integration of a metal-powder–PDMS thermal conduction block, thermoelectric cooler (TEC)-based temperature control, and a self-pressurized gas-diffusion micropump—significantly simplifying system architecture. The platform requires only one heat source to establish a complete three-temperature-zone thermal cycling profile. By leveraging the trapezoidal geometry of the PDMS block and tuning the copper-powder doping ratio, precise passive control over the denaturation, annealing, and extension stages is achieved, substantially reducing hardware complexity and cost while enhancing system robustness.

The self-pressurized gas-diffusion micropump operates without external pumps or valves, relying instead on the high flow resistance of capillary quartz tubes and a constant pressure differential within a sealed chamber to achieve pulse-free, stable sample flow over extended durations—sufficient for 40 PCR cycles. This passive driving mechanism not only eliminates the risk of mechanical failure but also enables practical implementation in disposable, closed microfluidic chips. Although amplification efficiency is slightly lower than that of commercial instruments (approximately 75–80%), likely due to factors such as nonspecific adsorption to microchannel walls, suboptimal residence times, and thermal crosstalk, further improvements are attainable through surface modification or thermal shielding strategies.

Notably, the TEC-based portable system (727 g, 6 W) operates solely on a 3.6 V lithium battery, making it suitable for field use or resource-limited settings. The overall design overcomes the traditional reliance of PCR on mains power and complex peripheral equipment, advancing point-of-care (POCT) molecular diagnostics toward lighter weight and higher integration. Future work will focus on integrating sample preparation, enabling multiplexed target detection, and developing smartphone-based readout systems to achieve a true “sample-in–answer-out” closed-loop diagnostic platform.

## 5. Conclusions

This chapter investigates a constant-temperature, single-heat-source continuous-flow PCR (CF-PCR) microdevice. Building upon the principles of continuous-flow PCR, we propose a CF-PCR approach driven by a single isothermal heating element combined with a simple temperature control module to achieve the complex thermal cycling required for PCR, resulting in a relatively straightforward control system. Moreover, by flexibly combining different thermal control modules, the system can be readily adapted to meet the specific temperature requirements of various PCR reagents.

Based on this approach, three microdevice configurations were developed: (1) an on-chip, single-heat-source, zone-heated PCR microdevice utilizing PDMS-based heat conduction; (2) an off-chip PCR microdevice also leveraging PDMS heat conduction; and (3) an off-chip PCR microdevice based on the thermoelectric cooling (TEC) Peltier effect. Furthermore, the TEC-based off-chip design was integrated into a compact, handheld PCR prototype, successfully achieving miniaturization and low power consumption.

CF-PCR is inherently amenable to integration with upstream (e.g., sample preparation) and downstream (e.g., detection) functions, making it particularly suitable for all-in-one portable PCR platforms. The choice of microdevice type can be tailored according to the target analyte and desired level of integration, with corresponding selections of materials and fabrication methods made to meet specific application requirements.

## Figures and Tables

**Table 1:** Upper surface temperature of PDMS block mixed with different proportions of copper powder.

Copper Powder Concentration (%)	0	2	4	6	8	10	15	18	20	Pure Copper
Top surface temperature (°C)	58	61.5	62.5	65.5	70	71	72.5	74.5	75	95

**Table 2:** Equipment.

Equipment	Manufacturer	Purpose Description
Gene Amplifier	Bioer Technology (Hangzhou)	Used as a commercial control for comparing amplification performance
Gel Electrophoresis System	Liuyi Biotechnology (Beijing)	Used for PCR product detection (gel electrophoresis)
Thermal Infrared Camera	Fortric	Used to monitor temperature distribution across different regions of the microdevice
CNC Precision Engraving Machine	Guangzhou Yubang	Used to fabricate microchannels on PMMA chips
Vacuum Drying Oven	Shanzhi Instruments (Shanghai)	Used for PDMS curing (70 °C, 1 h)
Ultrasonic Cleaner	Kemeng Electric (Guangzhou)	Used for cleaning PMMA chips (pre-bonding treatment)
Hot Press	Jingchuang Pneumatic Equipment (Shenzhen)	Used for thermal bonding and sealing of PMMA chips
Electronic Balance	Lichen Tech (Shanghai)	Used for weighing reagents such as BSA (Bovine Serum Albumin)
Micropipette	Dragon Lab (Beijing)	Used for sample loading (e.g., adding 40 μL of sample into a syringe)

**Table 3:** Core biological reagents.

Consumables and Reagents	Manufacturer	Description of Use
H7N9 avian influenza plasmid	Genewiz	Template DNA for amplification and verification
pGEM-3Zf (+) plasmid	Genewiz	Template DNA
Human papillomavirus (HPV) plasmid/Rubella virus (RUBV) plasmid+	(Implicitly procured from Genewiz)	Used for testing portable devices
Corresponding primers (H7N9, pGEM, HPV, RUBV)	Genewiz	PCR amplification primers
TaKaRa Premix Taq (Ex Taq)	TaKaRa	Core enzyme master mix for PCRs
10× TBE Buffer	Phygene Biotechnology	Diluted to 1× TBE for electrophoresis
DL2000 DNA Marker	TaKaRa	Molecular weight standard for electrophoresis
6× DNA Loading Buffer	Beijing Jialan	Loading dye for electrophoresis
Gel-Green	Jiangsu KeyGEN BioTECH	Nucleic acid stain (EB alternative)
Sterile Double Distilled Water	Phygene Biotechnology	For solution preparation

**Table 4:** Microfluidic Chip and Device Materials.

Consumables and Reagents	Manufacturer	Description of Use
Polydimethylsiloxane (PDMS)	DOW Chemical	Fabrication of thermal blocks (mixed with copper powder)
Curing agent	DOW Chemical	PDMS curing
PMMA sheets	Wenzhou Jiujun	Substrate material for on-chip PCR devices
PTFE tubing (inner diameter: 0.3 mm)	Shanghai ShenHui	Wrapped around PDMS as microfluidic channels
Fused silica capillary tubes (inner diameter: 25 μm, length: 10–15 cm)	(Manufacturer not listed, but classified as consumable)	Flow-resistance element in self-pressurized micropumps (critical!)
Bovine Serum Albumin (BSA)	AMEKO	Passivation of microchannels to reduce non-specific adsorption
Syringes (10 mL)	Guangzhou Cofoe Medical Devices	Construction of self-pressurized sample loading system
Needles (27G)	Jiaxing Huatai Yu	Connection of microfluidic tubing

**Table 5:** Temperature control and electronic components.

Consumables and Reagents	Manufacturer	Description of Use
Heating pads	Shanghai PTC	Single heat source (95 °C or 120 °C)
Thermoelectric cooling modules (TEC1-12712 or TEC2-25416)	China	Peltier-effect-based temperature control
Platinum resistance temperature sensor (PT1000)	China	Temperature feedback (potentially used in temperature control loop)
Temperature controller (TCM-M207)	China	Controls heating pad temperature (basic temperature control module)
Heat sinks + fans	Miaoxin/CYJ	Dissipate heat from the hot side of TECs (in portable devices)
Lithium battery (3.7 V, 12,000 mAh)	(Not listed, but part of power module)	Power supply for portable PCR device

**Table 6:** The primer sequence and fragment length needed for the experiment.

Name	Sequence (5′–3′)	Amplicon Length (bp)
H7N9	Fw TAC AGA CAA TCC CCG ACC GA	116
avian influenza	Rv GCC AAG TGT TAG CCC CAT CC	
	Fw CCG GCG AAC GTG GCG AGA AAG	137
pGEM-3Zf(+)	GAA GGG AAG AAA GC	
plasmid vector	Fw GCC AAC CCC TCC AGA AAC A Rv	
	CCC ACC TCC ACC AGT AAA CG	
Human	Fw GCC AAC CCC TCC AGA AAC A Rv	75
papillomavirus	CCC ACC TCC ACC AGT AAA CG	
RUBV	Fw ATT GTT ATG TAT GAG CGG TGA A	88
	Rv TTG TAA AGC CCT ATG AGT GAG C	

**Table 7:** The ratio of PCR solution.

Reagent	Volume (μL)
Premix Taq (Ex Version 2.0 plus dye)	10
Bovine Serum Albumin V	3
Sterile Double-Distilled Water	3
Sample DNA	2
Forward Primer (Fw)	1
Reverse Primer (Rv)	1

**Table 8:** The temperature of low-temperature zone under different voltage and plate material.

Voltage (V)	Temperature (°C)		
	92% Alumina Ceramic	Silicon Carbide Ceramic	304 Stainless Steel
1	30	30	34
1.5	34	32	36
2	38.5	35	37
2.5	48	46	47
3	56	54	56
3.2	58	57	60
3.4	60	62	63
3.6	63	65	67
3.8	68	69	72
4	70	73	75

## Data Availability

The data underlying this study contain sensitive or confidential information and cannot be made publicly available due to ethical or privacy constraints.
